# Effects of ‘4P’ Theory–Based Training on Innovative Capability in Clinical Nurses: A Pre–Post Study

**DOI:** 10.1155/jonm/2704124

**Published:** 2026-09-02

**Authors:** Shuiyu Zhang, Qiyu Li, Manzhen Bao, Miao Zhang, Qianyun Zhao, Xue Zhang, Xiyao Yang

**Affiliations:** ^1^ School of Nursing, Anhui Medical University, 15 Feicui Road, Hefei 230601, Anhui, China, ahmu.edu.cn; ^2^ Department of Nephrology, The Second Affiliated Hospital of Anhui Medical University, 678 Furong Road, Hefei 230601, Anhui, China, ahmu.edu.cn; ^3^ Nursing Department, The Second Affiliated Hospital of Anhui Medical University, Hefei 230061, China, ahmu.edu.cn

**Keywords:** 4P theory, innovation capability, nursing innovation, pre–post study

## Abstract

**Objective:**

To evaluate the effectiveness of a structured innovation training program based on the ‘4P’ theory (Person, Process, Press and Product) in enhancing the innovation capability of clinical nurses.

**Methods:**

We conducted a 1‐year training program with 122 nurses at The Second Affiliated Hospital of Anhui Medical University. In this single‐group pretest–posttest quasi‐experimental study, data were collected before and after the intervention using questionnaires and analysed with paired‐sample *t*‐tests.

**Results:**

Compared to the pretraining period, the posttraining scores for innovation capability among all participants, including innovative person, innovative process and innovative press, showed a significant increase (*p* < 0.05), while no statistically significant difference was observed in the innovative product score (*p* < 0.05). Repeated measures analysis of variance revealed that the improvement in posttraining innovation capability scores was significantly greater in the high‐efficiency innovation group (Δ = 8.10 ± 1.74) than in the proactive innovation group (Δ = 5.90 ± 3.29) (*F* = 21.309, *p* < 0.05). However, the improvement in posttraining innovative press scores was significantly higher in the proactive innovation group (Δ = 2.92 ± 5.23) compared to that in the high‐efficiency innovation group (Δ = 0.46 ± 5.67) (*F* = 11.699, *p* < 0.05).

**Conclusion:**

This study demonstrates that an innovation training strategy based on the ‘4P’ theory effectively enhances the innovation capability of clinical nurses. This is achieved by systematically stimulating individual innovative potential, providing methodological training, creating a supportive environment and promoting outcome translation, thereby validating the clinical applicability of this theory.

## 1. Introduction

Global shifts in disease burden and rising health demands are increasing the challenges nurses face when caring for patients with diverse backgrounds and conditions, often necessitating innovative approaches to problem‐solving [1]. Numerous national policies [2–4] have called for strengthening innovation within nursing, enhancing nurses’ innovation capability and further improving the quality and level of nursing care. Nursing innovation capability refers to nurses’ ability to proactively develop and implement new methods, technologies and tools—through teamwork and supportive channels—to promote health, prevent disease and improve patient outcomes [5].

Studies indicate that enhancing nurses’ innovation capability can improve patient experiences, reduce length of hospital stay, optimise resource utilisation and potentially lower mortality rates [6, 7]. It also facilitates the integration of nursing theory and practice, promoting continuous progress in nursing and healthcare [8]. Therefore, strengthening nurses’ innovation capability is urgently needed to meet growing demands for high‐quality care.

Although nurse‐led innovations can improve both patient outcomes and health systems, levels of innovation capability vary worldwide. Polster et al. [9] surveyed 217 clinical nurses in a Chicago hospital and found that 90.3% demonstrated strong innovation capability. Similarly, Stilgenbauer et al. [10] reported through a survey by the American Organization of Nurse Executives that clinical experts and nurse managers exhibited higher innovation levels than staff nurses. In Pakistan, Masood et al. [11] evaluated 587 nurses and 164 nurse managers and found an above‐average mean innovation score of 3.95 ± 0.35.

China accounts for approximately 29% of the world’s nursing workforce. However, its nursing innovation capability lags behind that reported in other countries. A nationwide survey [12] revealed that the average innovation score among Chinese nurses was 137.96 ± 21.16 (out of a maximum of 205), indicating a moderate to low level of innovation capability.

Several countries, including the Netherlands and Sweden, have implemented support systems such as FabLabs [13], workshops [14] and medical maker spaces [15] to provide nurses with access to innovation resources like 3D printers and laser cutters, effectively enhancing their innovation capability [16]. In China, however, research on nursing innovation remains largely focused on cross‐sectional surveys. Although many nurses express strong interest in innovation training [17], there is a scarcity of interventional studies aimed at cultivating and evaluating innovation capability.

Currently, widely applied foundational theories for enhancing clinical nursing innovation capability include Social Exchange Theory, the Componential Theory of Creativity, the Theory of Inventive Problem Solving (TRIZ) and the ‘4P’ Theory (Person, Process, Press and Product) [18]. Social Exchange Theory conceptualises the relationship between organisations and employees as a reciprocal social exchange [19]. When an organisation provides a supportive environment, employees perceive this and tend to reciprocate with higher organisational commitment and innovative behaviours. This theory underscores the role of the external organisational environment in shaping nurses’ innovative actions. The Componential Theory of Creativity posits that individual creativity emerges from the confluence of domain‐relevant expertise, creative thinking skills and intrinsic task motivation [20]. It highlights intrinsic motivation as the most critical component, with external environments ultimately influencing creativity by either enhancing or diminishing this internal drive. The Theory of Inventive Problem Solving, commonly known as TRIZ, represents a systematic and structured methodological theory for innovation [21]. Derived from the analysis of numerous patents, it summarises patterns of technical system evolution and general principles for resolving engineering contradictions, constituting essentially a methodology for innovation.

Proposed by Rhodes and later refined by Tardif and Sternberg (1993), the ‘4P’ Theory has become a classic theory in innovation research and has laid a foundation for subsequent studies [22]. It encompasses four interrelated elements: Person, Process, Press and Product. ‘Person’ refers to an individual’s positive psychological disposition toward creative activities, which is a vital component of creativity. ‘Process’ emphasises the cognitive and thinking pathways involved in creativity, focusing on creative thinking. ‘Press’ denotes the environment that either facilitates or hinders innovative work, highlighting the interaction between the individual and their context and analysing environmental factors that promote or inhibit innovation. ‘Product’ represents the tangible or intangible outcomes of innovation, including novel ideas, inventions, discoveries and theories.

Unlike single‐dimensional theories, the ‘4P’ theory provides a comprehensive perspective that captures the key elements influencing individual or team innovation capability, which aligns well with the inherently multidimensional nature of nursing practice. Existing research has indicated that nursing innovation is shaped by multiple interacting factors, including individual characteristics, thought processes, organisational environment and outcome orientation [23]. Single‐dimensional theories, which typically focus on only one of these aspects, struggle to address the reality that nursing innovation occurs within complex clinical settings. The integrated perspective of the ‘4P’ theory enables training programs to simultaneously address the individual, process, environment and product, thereby bridging the gap between innovative potential and clinical application. This makes it a robust strategic foundation for designing training programs aimed at cultivating innovative competence.

**FIGURE 1:**
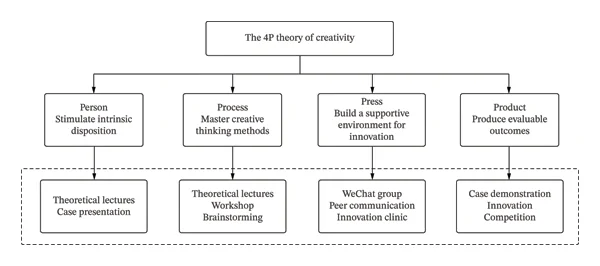
A conceptual flow diagram of the 4P training framework. Note: This figure illustrates the four components (Person, Process, Press and Product) and their corresponding training activities, including theoretical lectures, case presentations, workshops, brainstorming, WeChat group communication, peer communication, innovation clinic, case demonstration and innovation competition.

Therefore, grounded in the ‘4P’ theory, this study formulates a nurse innovation training strategy centred on its four core components: (1) stimulating nurses’ intrinsic inclination toward innovation through theoretical courses and case demonstrations; (2) enhancing creative thinking and problem‐solving abilities via lectures, workshops and brainstorming sessions to master innovative thinking methods; (3) building a supportive environment by establishing innovation clinics, WeChat groups and peer‐exchange platforms; and (4) showcasing innovative products through case presentations and innovation competitions, with products evaluated based on novelty, practicality and completeness. Accordingly, the practical purpose of this study was twofold: (1) to enhance clinical nurses’ overall innovation capability, so that they are able to apply creative thinking to solve problems in daily practice; and (2) to strengthen their performance across the four innovation dimensions, enabling them to drive meaningful improvements in patient care and clinical workflows. A conceptual flow diagram of the 4P theory‐based training framework is presented in Figure 1.

## 2. Methods

### 2.1. Design and Context

This study was conducted at the Second Affiliated Hospital of Anhui Medical University in Hefei, Anhui Province, China, from July 2023 to July 2024, employing a single‐group pretest–posttest quasi‐experimental design. Participants received a 1‐year innovation training programme after enrolment, with evaluations performed at two time points: before and after the training. This study was reported in accordance with the TREND (Transparent Reporting of Evaluations with Nonrandomized Designs) statement.

### 2.2. Participants

Recruitment invitations were distributed via Questionnaire Star, and interested nurses were instructed to contact the research team for further details. Eligible participants underwent individual baseline assessments using standardised questionnaires. Upon completion of the 1‐year intervention programme, participants were reevaluated using the same instruments.

To capture variations in innovation capability and reflect the progressive impact of the training, an initial survey was administered via SurveyStar to assess nurses’ innovation needs and motivations, along with collecting data on the number of patents they had applied for. We selected the number of patents—an objective and verifiable output metric—as the criterion for subgroup classification, with the aim of clearly distinguishing between different stages of nurses’ engagement in innovative practice. Based on patent output, nurses were categorised into three groups: a routine execution group (no patents), a proactive innovation group (applying for or holding one patent) and a high‐efficiency innovation group (holding two or more patents). However, due to challenges in meeting recruitment targets for the routine execution group and considering practical constraints in implementing the intervention, only participants from the proactive innovation group and high‐efficiency innovation group were included in the study. To ensure research feasibility and optimise resources, the final study sample included only participants from the proactive innovation group and the high‐efficiency innovation group. This methodological decision to adjust the sampling strategy based on practical considerations is justified and aligns with the approach adopted by Sun et al. [24] in their study. Eligibility criteria required participants to hold a valid nursing qualification certificate, be able to commit to the training schedule and provide consent to participate. Exclusion criteria included not being employed at the affiliated hospital during the study period, enrolment in other training programmes or concurrent studies and being an intern or visiting nurse.

### 2.3. Ethical Considerations

This study was approved by the IRB of the Second Affiliated Hospital of Anhui Medical University (No. SL‐YX [YS] 2023‐035). All participants gave written informed consent after being fully informed of the training benefits and intervention details, agreeing to participate and to have their data used for research.

### 2.4. Sample Size

The sample size was calculated based on nursing innovation capability as the primary outcome measure. According to previous reports in similar studies [25], the following parameters were used: *δ* = 15.22, *S* = 33.4, *α* = 0.05, *β* = 0.1, power (1–*β*) = 0.9, Zα/2 = 1.96 and Zβ = 1.28. Using the formula for continuous outcomes, a minimum of 102 participants was initially estimated. Accounting for a potential dropout rate of 20%, the final required sample size was determined to be at least 122 participants.

### 2.5. Statistical Analysis

Statistical analyses were performed using SPSS version 26.0. Continuous normally distributed variables were expressed as mean ± standard deviation (x̄ ± *s*) and categorical variables as frequencies and percentages. Between‐group comparisons of baseline characteristics were conducted using chi‐squared tests and independent two‐sample *t*‐tests. Pre‐ and postintervention effects were analysed using paired‐sample *t*‐tests. A repeated‐measures ANOVA was conducted to compare the magnitude of improvement between the two groups after the intervention. Because the repeated‐measures ANOVA involved only two time points (baseline and postintervention), the sphericity assumption was automatically satisfied; therefore, Mauchly’s test of sphericity was not performed. A *p* value of less than 0.05 was considered statistically significant.

### 2.6. Intervention

The nurse innovation training strategy was developed based on the ‘4P’ theory, encompassing four core dimensions: Person, Process, Press and Product. The specific interventions are as follows: (1) Person: focusing on nurses’ personal traits and affective attitudes to stimulate their intrinsic inclination toward innovation. (2) Process: equipping nurses with innovative thinking methods to enhance creative thinking and problem‐solving abilities. (3) Press: building a supportive environment that encourages innovative practices among nurses. (4) Product: evaluating innovative outcomes based on novelty, practicality and completeness. Detailed information regarding the nurse innovation training strategy is presented in Table 1.

**TABLE 1:** Specific strategies for cultivating nurse innovation capability.

4P theory components	Training activities	Duration
Person	(1) Kick‐off meeting: clarify objectives, showcase cases, stimulate innovation motivation. (2) Tiered instruction: innovation culture and basic concepts to strengthen cognitive foundations of the two nurse subgroups.	2 credit hours (Months 1–2)

Process	(1) Instruction on innovative thinking and methodologies. (2) Instruction on patent application, ethics and research translation basics. (3) Interdisciplinary workshops to develop innovation proposals under mentor guidance. High‐efficiency innovation group: additional advanced tasks to iteratively refine solutions and evaluate translational potential.	8 credit hours (Months 3–10)

Press	(1) ‘Innovation Clinic’: monthly one‐on‐one expert consultation (2) WeChat group: daily Q&A, knowledge sharing, peer exchange	(1) Monthly (Months 1–12) (2) Ongoing (Months 1–12)

Product	(1) Innovation exhibition and competition: Proactive innovation group presented case studies; high‐efficiency innovation group participated in contests (2) Expert evaluation to promote research translation	2 credit hours (Months 11–12)

The nurse innovation cultivation strategy employed a blended online–offline format delivered over 12 months. The offline component comprised 12 credit hours of structured training activities (1 credit hour = 60 min). Table 1 presents the detailed training activities and their distribution across the intervention period. Two chief nurses served as project leads, responsible for defining core topics, allocating organisational resources and overseeing the design and implementation of the intervention. Four associate chief nurses, all holding relevant teaching qualifications, delivered the theoretical instruction. Seven nurse supervisors managed online content and organised activities such as innovation competitions. Three postgraduate nursing students and one undergraduate student were responsible for data collection and analysis. All team members had completed prior innovation training workshops. The online component offered continuous guidance and peer interaction through a dedicated WeChat group throughout the 12‐month intervention period.

### 2.7. Measurements

The research team developed a questionnaire using Questionnaire Star, which participants completed online via WeChat groups at baseline and 1 year after the intervention. Nurse innovation capability was assessed using the Nurse Innovation Ability Scale (NIAS) [5]. The scale consists of 41 items across four dimensions corresponding to the 4P theory: Person (17 items), Process (12 items), Press (7 items) and Product (5 items). A five‐point Likert scale is used, ranging from 1 (*very unfit*) to 5 (*very fit*), with higher total scores indicating greater innovation capability. The original validation study [5] reported Cronbach’s *α* of 0.938 for the overall scale. In the present study, the overall scale demonstrated excellent internal consistency, with Cronbach’s *α* of 0.943. Cronbach’s *α* values for the four subscales were 0.917 for Person, 0.925 for Process, 0.940 for Press and 0.886 for Product. Sociodemographic data—including gender, age, marital status, department, years of experience, professional title and education level—were also collected at baseline. Additionally, the number of patents and publications held by participants was collected via the questionnaire. Participants were required to provide official documentation for the reported patents and publications, which were subsequently verified by the research team.

## 3. Results

### 3.1. Baseline Characteristics of Participants

A total of 156 participants initially agreed to participate in the study. Of these, 21 were excluded for not meeting the inclusion criteria, and 13 did not complete the programme due to insufficient compliance preventing final assessment, or changes in family or health circumstances unrelated to the study. The final sample consisted of 122 participants (6 males, 116 females; mean age 37.22 ± 5.50 years), with 61 assigned to the proactive innovation group and 61 to the high‐efficiency innovation group.

Baseline characteristics are presented in Table 2. Apart from work experience and professional title, no statistically significant differences were observed between the two groups (*p* > 0.05). However, significant differences were identified in patent acquisition, paper publication and overall innovation capability (*p* < 0.05). No significant intergroup differences were found in the innovative press or innovative product dimensions (*p* > 0.05).

**TABLE 2:** Comparison of baseline characteristics between the proactive innovation group and the high‐efficiency innovation group.

Feature	Proactive innovation group (*n* = 68)	High‐efficiency innovation group (*n* = 67)	*p* value
General information			
Female, *n* (%)	66 (97.1%)	62 (92.5%)	0.274
Age (years), average (standard deviation)	36.69 ± 7.54	37.23 ± 4.79	0.624
Marital status			0.089
Single, *n* (%)	14 (20.6%)	5 (7.50%)	
Married, *n* (%)	52 (76.5%)	60 (89.6%)	
Divorced or widowed, *n* (%)	2 (2.90%)	2 (2.90%)	
Department			0.134
Internal medicine, *n* (%)	16 (23.5%)	11 (16.4%)	
Surgery, *n* (%)	28 (41.2%)	20 (29.9%)	
Obstetrics and paediatrics, *n* (%)	2 (2.9%)	2 (3.0%)	
Emergency and outpatient, *n* (%)	7 (10.3%)	5 (7.5%)	
Intensive care unit, *n* (%)	15 (22.1%)	29 (43.3%)	
Years of work experience			0.026
≤ 10 years, *n* (%)	23 (33.8%)	12 (17.9%)	
11–20 years, *n* (%)	40 (58.8%)	39 (58.2%)	
21–30 years, *n* (%)	4 (5.9%)	12 (17.9%)	
≥ 31 years, *n* (%)	1 (1.5%)	4 (6.0%%)	
Job title			0.032
Nurse, *n* (%)	19 (27.9%)	7 (10.4%)	
Senior nurse, *n* (%)	41 (60.3%)	48 (71.6%)	
Deputy chief nurse, *n* (%)	8 (11.8%)	12 (17.9%)	
Academic credentials			0.693
Junior college diploma, *n* (%)	3 (4.4%)	3 (4.5%)	
Bachelor, *n* (%)	63 (92.6%)	60 (89.6%)	
Master’s degree or higher, *n* (%)	2 (2.9%)	4 (6.0%)	
Number of patents obtained, mean (standard deviation)	1.0 ± 0.00	3.06 ± 1.54	< 0.01
Number of published papers, mean (standard deviation)	2.00 ± 1.90	2.82 ± 0.35	0.04
Total innovation capability score, mean (standard deviation)	138.06 ± 17.38	145.48 ± 20.07	0.023
Innovative person, mean (standard deviation)	55.63 ± 7.36	60.39 ± 9.21	0.001
Innovative process, mean (standard deviation)	39.63 ± 5.79	42.07 ± 7.22	0.032
Innovative press, mean (standard deviation)	26.66 ± 5.25	27.25 ± 5.58	0.526
Innovative product, mean (standard deviation)	16.13 ± 3.40	15.76 ± 3.29	0.520

*Note:* Baseline characteristics were assessed in the enrolled sample (*n* = 68 in the proactive innovation group and *n* = 67 in the high‐efficiency innovation group). The final analytic sample for pre‐ and postintervention comparisons was *n* = 61 per group, as detailed in Section 3.1.

We further examined the item‐level scores within each group. In the proactive innovation group, the three highest‐scoring items—one from the innovative person dimension and two from the innovative press dimension—were as follows: mastery of patient safety knowledge (4.03 ± 0.75), support from hospital leadership (3.94 ± 0.83) and participation in hospital‐based innovation training (3.93 ± 0.80). The three lowest‐scoring items, all within the innovative person dimension, were the ability to read literature in foreign languages (2.09 ± 0.75), mastery of basic statistical knowledge (2.32 ± 0.80) and understanding of experimental research techniques (2.74 ± 0.82). In the high‐efficiency innovation group, the top three items—representing the innovative person, innovative process and innovative product dimensions—were active willingness to learn (4.07 ± 0.72), effective communication between medical staff and patients (4.07 ± 0.80) and acquisition of patents for innovative ideas (3.97 ± 0.89). The lowest‐scoring items—two from the innovative person dimension and one from the innovative product dimension—were the ability to read foreign language literature (2.42 ± 1.00), mastery of basic statistical knowledge (3.36 ± 0.87) and clinical application of innovative products (2.57 ± 0.94).

### 3.2. Intervention Effects

The innovation capability outcomes for all participants (*n* = 122), assessed at baseline and after the 1‐year intervention, are presented in Table 3. Statistically significant improvements were observed across all dimensions (*p* < 0.05) except for innovative product. Specifically, the cumulative number of patents increased by 37.8% (from 251 by the end of 2023 to 346 by the end of 2024), and the cumulative number of published papers increased by 39.2% (from 286 by the end of 2023 to 398 by the end of 2024).

**TABLE 3:** Changes in innovation capability scores for all participants (*n* = 122).

Item	Pretest, M ± SD	Posttest, M ± SD	Change (posttest–pretest), M ± SD	Confidence interval (95% CI)	*T* value	*p* value
Total innovation Capability score	140 ± 17.50	147 ± 17.02	7.00 ± 2.84	(6.49.7.51)	27.224	< 0.001
Innovative person	57.43 ± 8.39	60.19 ± 8.8	2.76 ± 5.08	(1.85.3.67)	6.003	< 0.001
Innovative process	40.49 ± 6.51	42.64 ± 6.44	2.15 ± 4.26	(1.38.2.91)	5.564	< 0.001
Innovative press	27.05 ± 5.22	28.28 ± 3.47	1.23 ± 5.69	(0.21.2.25)	2.387	< 0.019
Innovative product	15.88 ± 3.26	16.09 ± 3.30	0.21 ± 4.03	(‐0.51.0.94)	0.584	0.561
number of patents obtained	2.06 ± 1.56	2.84 ± 2.03	0.78 ± 0.69	(0.66.0.90)	12.529	< 0.001
Number of published papers	2.34 ± 2.34	3.26 ± 4.49	0.92 ± 2.59	(0.45.1.38)	3.911	< 0.001

Descriptive statistics and repeated‐measures ANOVA results for the proactive innovation group (*n* = 61) and the high‐efficiency innovation group (*n* = 61) are presented in Table 4. A significant time effect was observed across all dimensions in both groups (*p* < 0.05). Significant time × group interaction effects were found for the total innovation capability score, innovative press and the number of published papers (*p* < 0.05). Specifically, the improvement in the total innovation capability score and the number of published papers was more pronounced in the high‐efficiency innovation group, whereas the improvement in innovative press was greater in the proactive innovation group.

**TABLE 4:** Repeated‐measures ANOVA results and descriptive statistics for innovation capability scores before and after the intervention (*n* = 61 per group).

Item	Pretest (M ± SD)	Posttest (M ± SD)	Time effect			Interaction effect		
	Proactive/high‐eff.	Proactive/high‐eff.	*F*	*p*	Partial *η* ^2^	*F*	*p*	Partial *η* ^2^
Total innovation capability score	126.67 ± 11.18/153.33 ± 9.49	132.57 ± 8.89/161.43 ± 9.23	113.240	< 0.001	0.485	14.239	< 0.001	0.106
Innovative person	51.46 ± 5.82/63.39 ± 5.98	53.39 ± 5.31/66.98 ± 5.94	36.716	< 0.001	0.234	3.298	0.072	0.027
Innovative process	35.89 ± 4.15/45.10 ± 5.02	37.30 ± 3.26/47.98 ± 3.87	31.655	< 0.001	0.209	3.735	0.056	0.030
Innovative press	25.00 ± 5.22/28.64 ± 3.80	27.92 ± 3.11/29.10 ± 4.38	6.203	0.014	0.049	11.699	0.001	0.089
Innovative product	14.16 ± 2.66/16.92 ± 3.13	14.84 ± 3.06/18.02 ± 2.72	6.126	0.015	0.049	0.355	0.552	0.003
number of patents obtained	1.00 ± 0.00/3.11 ± 1.59	1.69 ± 0.47/3.98 ± 2.33	158.426	< 0.001	0.569	2.124	0.148	0.017
Number of published papers	0.79 ± 0.66/3.90 ± 2.35	1.16 ± 0.76/5.36 ± 5.50	15.870	< 0.001	0.117	5.511	0.021	0.044

*Note:* Pretest and posttest columns present means ± standard deviations for the Proactive and High‐Eff groups, with Proactive values listed first before the slash and High‐Eff values after the slash. Within‐group comparisons (paired *t*‐test results) are provided in Supporting Table S1.

## 4. Discussion

This study adopted an innovation cultivation strategy based on the ‘4P’ theory to enhance the innovation capability of clinical nurses. Following the training, nurses’ innovation capability scores improved significantly (*p* < 0.05), which is consistent with findings from similar quasi‐experimental studies by Gao et al. [26], Li et al. [27] and Liu et al. [28] that aimed to evaluate theory‐driven training for improving nurses’ innovation capability. According to the ‘4P’ theory, achieving effective improvement in innovation capability requires the dynamic interaction of all four elements—Person, Process, Press and Product—rather than focusing on a single dimension [22]. The reasons for the enhancement of nurses’ innovation capability in this study can be analysed as follows.

First, ‘Person’ is the starting point of innovative behaviour. The nurses recruited for this study had already demonstrated proactive innovative behaviour and possessed insight and foresight regarding challenges in clinical practice [29]. However, motivation must be translated into concrete actions to generate practical outcomes, a process that relies on the systematic support of the ‘Process’ component.

Second, the ‘Process’ component translates motivation into structured action. By systematically delivering methodological instruction, workshops and facilitating brainstorming sessions, this study provided nurses with structured skills and supportive opportunities, thereby transforming motivation into actionable efforts. Scholars have pointed out that skills constitute the competency system of innovation and can cultivate and stimulate individual innovative thinking. The internalisation of skills through hands‐on training further facilitates the translation of innovative ideas into tangible outcomes [30]. Workshops, in particular, have been widely adopted and proven effective in both domestic and international training programmes [31, 32].

Third, ‘Press’ plays a supportive and moderating role. At present, many clinical nurses encounter barriers in accessing relevant resources and technical support during the innovation process, which limits their innovative potential [33]. By establishing an ‘Innovation Clinic’ and a dedicated WeChat group, this study provided professional guidance and real‐time communication channels, which not only enhanced nurses’ willingness to participate but also improved the effectiveness of skills training. Conversely, nurses’ active engagement in the ‘Process’ can prompt management to provide recognition and allocate resources favourably, further optimising ‘Press’ and realising mutual reinforcement among the three components.

Finally, the presentation and application of ‘Product’ can also exert a feedback effect on ‘Person’, ‘Process’ and ‘Press’. Innovation outcomes help to enhance nurses’ self‐efficacy, thereby strengthening their intrinsic motivation [34]. Exemplary cases serve as models that help to optimise ‘Press’ and prompt ‘Person’ to re‐evaluate their own innovation potential. The feedback relationships described above indicate that the interactions among the four components are not unidirectional causal relationships but are instead mutually influential.

In the Clinical Nurse Innovation Ability Scale developed by scholar Yan Jianjun, the ‘Innovative Product’ dimension refers to the tangible outcomes generated when clinical nurses translate innovative ideas into practice. This includes patent applications, academic publications, product translation and clinical implementation [5]. While the change in the Innovative Product dimension was not statistically significant for all participants (*p* > 0.05), further analysis revealed that the score in the high‐efficiency innovation group increased significantly after training (*p* < 0.05), whereas no significant change was observed in the proactive innovation group (*p* > 0.05). This difference may be attributed to the distinct training strategies employed for each group. The innovation competition designed for the high‐efficiency innovation group had clear goal orientation and output requirements. Its structured process and evaluation criteria directly motivated nurses to develop well‐structured and concrete innovative outcomes. In contrast, the case‐sharing approach used for the proactive innovation group emphasised inspiration and experience exchange. The resulting outcomes were often at an exploratory stage and might not have fully met the Innovative Product criteria of the scale in terms of completeness. Scholars have also highlighted that systematic methodological training, ongoing guidance from an innovation mentoring team, and continuous pathway support are essential throughout the process of product translation and clinical implementation to sustainably promote the transformation of clinical nursing innovations and technological advances [35].

The study revealed a significant increase in the number of patents and publications within both groups. While these findings may be influenced by a time‐lag effect and thus cannot be solely attributed to the immediate impact of the training program, they nevertheless offer preliminary support for its positive role in enhancing participants’ engagement in innovative behaviours and their intention to generate tangible outcomes.

According to Rogers’ diffusion of innovation theory, innovation adopters can be categorised into ‘innovators’ and ‘early adopters’: innovators are risk‐taking and pursue breakthrough outcomes, whereas early adopters place greater emphasis on social recognition, organisational support and observable results [36]. In this study, nurses in the high‐efficiency innovation group had already generated multiple patents at baseline, and their behavioural characteristics align with the role of ‘innovators’. Nurses in the proactive innovation group, although having attempted innovation, had not yet produced stable outcomes, making them closer to the role of ‘early adopters’.

One empirical study found that innovators are primarily driven by intrinsic factors, while early adopters rely more on extrinsic factors [37]. This is consistent with the results of our study: after the intervention, the improvement in innovation capability in the high‐efficiency innovation group (Δ = 8.10 ± 1.74) was significantly greater than that in the proactive innovation group (Δ = 5.90 ± 3.29) (*F* = 21.309, *p* < 0.05). Conversely, the improvement in the innovative press dimension was significantly greater in the proactive innovation group (Δ = 2.92 ± 5.23) than in the high‐efficiency innovation group (Δ = 0.46 ± 5.67) (*F* = 11.699, *p* < 0.05).

Specifically, the strong goal‐oriented and highly challenging training components designed for the high‐efficiency innovation group, such as workshops and innovation competitions, significantly enhanced their innovation capability. This result may also be partially attributed to the longer working years and higher professional titles of nurses in this group, which suggest that they possessed stronger intrinsic motivation and richer practical experience prior to the intervention; the systematic and targeted training further promoted the improvement of their innovation competence. In contrast, early adopters rely more on signals from the organisational environment to confirm the safety and value of innovation. The innovation clinic and WeChat group interactions established in this training were positively perceived by nurses in the proactive innovation group, thereby significantly enhancing their positive perception of the innovative press. This positive perception, in turn, increased their trust in and willingness to adopt innovation.

The innovation cultivation strategy based on the ‘4P’ theory can effectively enhance nurses’ clinical innovation capability. However, this study also has several limitations: First, the use of a single‐group, pretest‐posttest quasi‐experimental design without a control group limits the ability to fully rule out the influence of maturation or history effects on the outcomes. Future studies would benefit from incorporating a control group to strengthen causal inference. Second, the primary outcomes relied on self‐report measures. Although anonymity was assured, responses may still be subject to social desirability bias, which could lead to some overestimation of the immediate training effects.

Third, this study has dual limitations regarding the sample and subgroup classification. On one hand, the final sample only included participants from the proactive innovation group and the high‐efficiency innovation group. Consequently, the findings are primarily applicable to nurses who already have a track record of innovation attempts, and whether the effects can be generalised to all nurses requires further investigation. On the other hand, when comparing these two subgroups, the classification was based solely on the baseline number of patents, without measuring individual innovation‐related traits such as creative self‐efficacy. Therefore, the observed between‐group differences might be confounded by these underlying characteristics. Furthermore, there were significant differences in multiple characteristics (e.g., working years, professional titles) between the two subgroups at baseline. These baseline differences may have, to some extent, influenced the magnitude of improvement observed after the intervention. Future studies are recommended to incorporate both innovation‐related traits and other relevant covariates in baseline assessments to better control for confounding factors.

Fourth, regarding the interpretation of outcomes, the observed increases in patents and publications are constrained by the inherent time required for their development and may reflect a time‐lag effect. Thus, they cannot be entirely attributed to the one‐year training program alone. Longer‐term follow‐up is suggested to better capture the sustained impact of the intervention. Finally, the study was conducted in a single tertiary hospital, which may limit the generalisability of the findings and introduce selection bias. Future multi‐centre studies are encouraged, incorporating contextual factors such as organisational innovation climate as moderators in the analysis. This would help evaluate the general applicability of the training program across different organisational settings and support the development of more tailored strategies to foster nursing innovation.

### 4.1. Implications for Nursing Management

Applying the ‘4P’ theory to nursing innovation education can significantly enhance the clinical innovation capability of nurses. This study found that nurses in different groups exhibited distinct patterns of improvement: the high‐efficiency innovation group showed a greater increase in innovation capability, whereas the proactive innovation group demonstrated more significant improvement in the innovative press dimension. This suggests that nursing managers should establish a stratified training system that matches differentiated strategies based on nurses’ levels of innovation output. Notably, the participants in this study were all selected based on their prior innovation output; they were already nurses actively engaged in innovative activities within the clinical setting. Through systematic training, these nurses not only enhanced their personal innovation capability but also have the potential to become innovation leaders at the clinical frontline. Nursing managers should prioritise the identification and focused cultivation of such talent, incorporating them into the development of an innovation talent echelon to accelerate the diffusion of nursing innovation within the organisation. Furthermore, nursing managers should integrate the ‘4P’ framework into nursing talent development programmes, promoting the deep integration of theory with clinical practice. Collaborative efforts among nursing administration departments, clinical units, and research/education institutions are essential for integrating resources and building a sustainable innovation education ecosystem. This requires a long‐term commitment to embedding innovation capability cultivation at the core of nursing team development, achieving systematic and routine innovation education, thereby enhancing the quality of nursing services and advancing the value of the nursing profession within the healthcare system.

## 5. Conclusion

This study confirms that the innovation cultivation strategy based on the ‘4P’ theory can effectively enhance nurses’ innovation capability, with the high‐efficiency innovation group and the proactive innovation group showing differentiated improvements in innovation capability and innovative press perception, respectively. This suggests that training should be designed in a tiered manner, thereby developing the ‘4P’ theory into a dynamically adaptable tool and providing a basis for nursing managers to establish a tiered support system and foster a sustainable culture of innovation.

## Author Contributions

Shuiyu Zhang and Qiyu Li contributed equally as co‐first authors. Study design: Xiyao Yang and Qiyu Li. Intervention implementation and data collection: Shuiyu Zhang, Manzhen Bao, Miao Zhang, Qianyun Zhao and Xue Zhang. Data analysis: Qiyu Li and Qianyun Zhao. Manuscript drafting: Shuiyu Zhang and Qiyu Li. Critical revision and final approval: all authors. Supervision and funding: Xiyao Yang.

## Funding

This work was supported by the Anhui Province Quality Engineering Project (Grant No. 2023jyxm1124).

## Disclosure

All authors agree to be accountable for the work.

## Consent

Written informed consent was obtained from all participants.

## Conflicts of Interest

The authors declare no conflicts of interest.

## Supporting Information

Additional supporting information can be found online in the Supporting Information section.

## Supporting information


**Supporting Information** Supporting Table S1. Within‐group comparisons (paired *t*‐test results).

## Data Availability

The datasets used and/or analysed during the current study are available from the corresponding author, Yang Xiyao, upon reasonable request.

## References

[1] Jimenez-Gomez M. A. , Cardenas-Becerril L. , Velasquez-Oyola M. B. , Carrillo-Pineda M. , and Barón-Díaz L. Y. , Reflective and Critical Thinking in Nursing Curriculum, Revista Latino Americana de Enfermagem. (2019) 27, 10.1590/1518-8345.2861.3173. PMC6896806 31826151

[2] National Health Talent Development , 14th Five-Year Plan: Building a Highland for Life and Health Talent, China Talent. (2022) no. 10.

[3] O′Hara S. , Ackerman M. H. , Raderstorf T. , Kilbridge J. F. , and Melnyk B. M. , Building and Sustaining a Culture of Innovation in Nursing Academics, Research, Policy, and Practice: Outcomes of the National Innovation Summit, Journal of Professional Nursing. (2022) 43, 5–11, 10.1016/j.profnurs.2022.08.001. 36496244

[4] Weatherford B. , Bower K. A. , and Vitello-Cicciu J. , The CNO and Leading Innovation: Competencies for the Future, Nursing Administration Quarterly. (2018) 42, no. 1, 76–82, 10.1097/naq.0000000000000263. 29194335

[5] Yan J. J. , Yang J. G. , Jiang Y. et al., Development of Nurse Innovation Ability Scale and Its Reliability and Validity Testing, Chinese Journal of Nursing. (2018) 53, no. 10, 1213–1217.

[6] Su J. J. and Yu D. S. , Effects of a Nurse-Led eHealth Cardiac Rehabilitation Programme on Health Outcomes of Patients With Coronary Heart Disease: A Randomised Controlled Trial, International Journal of Nursing Studies. (2021) 122, 10.1016/j.ijnurstu.2021.104040. 34333211

[7] Jiang Y. , Ramachandran H. J. , Teo J. Y. C. et al., Effectiveness of a Nurse-Led Smartphone-Based Self-Management Programme for People With Poorly Controlled Type 2 Diabetes: ARandomized Controlled Trial, Journal of Advanced Nursing. (2022) 78, no. 4, 1154–1165, 10.1111/jan.15178. 35170786

[8] Thomas T. W. , Seifert P. C. , and Joyner J. C. , Registered Nurses Leading Innovative Changes, Online Journal of Issues in Nursing. (2016) 21, no. 3, 10.3912/OJIN.Vol21No03Man03. 27856917

[9] Polster D. and Villines D. , An Exploratory Descriptive Study of Registered Nurse Innovation: Implications for Levels of Adoption, Clinical Nurse Specialist. (2017) 31, no. 1, E1–E9, 10.1097/NUR.0000000000000264. 27906736

[10] Stilgenbauer D. J. and Fitzpatrick J. J. , Levels of Innovativeness Among Nurse Leaders in Acute Care Hospitals, Journal of Nursing Administration. (2019) 49, no. 3, 150–155, 10.1097/NNA.0000000000000729. 30741776

[11] Masood M. and Afsar B. , Transformational Leadership and Innovative Work Behavior Among Nursing Staff, Nursing Inquiry. (2017) 24, no. 4, 10.1111/nin.12188. 28150910

[12] Yan J. J. , Yang J. G. , Jiang Y. et al., A National Survey of Innovation Capability Among Clinical Nurses in China, Japan Journal of Nursing Science. (2021) 36, no. 11, 52–55.

[13] Caroly S. , Barcellini F. , Barros M. , Catel A. , Nguyen H. M. , and Zwolinski P. , Different Forms of Fablab Organization and Their Impact on Collaboration and Innovation, Applied Ergonomics. (2025) 122, 10.1016/j.apergo.2024.104399. 39426368

[14] Shinohara M. , Nakamura T. , Kunikata N. , Okudera H. , and Kuroda Y. , A Half-Day Stroke Workshop Based on the Kirkpatrick Model to Improve New Clinical Staff Behavior, Journal of Advances in Medical Education and Professionalism. (2020) 8, no. 1, 10–17, 10.30476/jamp.2019.74874.0. 32039268 PMC6946945

[15] Svensson P. O. and Hartmann R. K. , Policies to Promote User Innovation: Makerspaces and Clinician Innovation in Swedish Hospitals, Research Policy. (2018) 47, no. 1, 277–288, 10.1016/j.respol.2017.11.006.

[16] Rigtering C. , Spaans L. J. , and de Jong J. P. J. , How to Bridge the Nurse Innovation-Diffusion Gap? an In-Depth Case Study of Create4Care, Frontiers in Public Health. (2023) 11, 10.3389/fpubh.2023.1209965. PMC10434511 37601181

[17] Yan D. , Li M. , Zhang Y. et al., A Qualitative Study of Facilitators and Barriers to Nurses’ Innovation at Work, Journal of Nursing Management. (2022) 30, no. 7, 3449–3456, 10.1111/jonm.13811. 36121750

[18] Hu W. T. , Jiang Y. , Wang Y. et al., Research Progress on Clinical Nursing Innovation Ability, Chinese Journal of Nursing Education. (2021) 18, no. 2, 178–182.

[19] Blau P. M. , Exchange and Power in Social Life, 1964, Wiley.

[20] Amabile T. M. , Schatzel E. A. , Moneta G. B. , and Kramer S. J. , Leader Behaviors and the Work Environment for Creativity: Perceived Leader Support, Leadership Quarterly. (2004) 15, no. 1, 5–32, 10.1016/j.leaqua.2003.12.003.

[21] Boavida R. , Navas H. , Godina R. , Carvalho H. , and Hasegawa H. , A Combined Use of TRIZ Methodology and Eco-Compass Tool as a Sustainable Innovation Model, Applied Sciences. (2020) 10, no. 10, 10.3390/app10103535.

[22] Sternberg R. J. and Lubart T. I. , Investing in Creativity, American Psychologist. (1996) 51, no. 7, 677–688, 10.1037/0003-066X.51.7.677.

[23] Xu D. , Influencing Factors and Developmental Needs of Nurses’ Innovative Ability, 2024, Bengbu Medical University.

[24] Sun T. , Xu Y. , Xie H. , Ma Z. , and Wang Y. , Intelligent Personalized Exercise Prescription Based on an eHealth Promotion System to Improve Health Outcomes of Middle-Aged and Older Adult Community Dwellers: Pretest-Posttest Study, Journal of Medical Internet Research. (2021) 23, no. 5, 10.2196/28221. PMC8185615 34028359

[25] Huang X. , Wang R. , Chen J. et al., Kirkpatrick’s Evaluation of the Effect of a Nursing Innovation Team Training for Clinical Nurses, Journal of Nursing Management. (2022) 30, no. 7, 2165–2175, 10.1111/jonm.13504. 34747090

[26] Gao L. , Lu Q. , Hou X. , Ou J. , and Wang M. , Effectiveness of a Nursing Innovation Workshop at Enhancing Nurses’ Innovation Abilities: A Quasi-Experimental Study, Nursing Open. (2022) 9, no. 1, 418–427, 10.1002/nop2.1080. 34687153 PMC8685873

[27] Li W. , Zhang Y. X. , Tang Q. H. et al., Application Effect of CDIO Model-Based Training Method in Cultivating Innovation Ability of Radiotherapy Department Nurses, Health Vocational Education. (2025) 43, no. 9, 58–62.

[28] Liu H. Y. , Wang I. T. , Chen N. H. , and Chao C. Y. , Effect of Creativity Training on Teaching for Creativity for Nursing Faculty in Taiwan: A Quasi-Experimental Study, Nurse Education Today. (2020) 85, 10.1016/j.nedt.2019.104231. 31765871

[29] Ma J. J. , Wang S. , Wei Y. L. et al., Mediating Effect of Workplace Spirituality Between Dualistic Work Stress and Proactive Innovation Behavior in Junior Nurses, Journal of Nursing. (2023) 30, no. 3, 68–73.

[30] Liu Y. , Research on Cultivation Strategies of Adolescents’ Innovative Literacy Based on Ecosystem Theory, China Education Technique and Equipment. (2025) no. 11, 1–4+19.

[31] Zhang Z. Y. , He X. F. , Xiang Q. et al., Application of Production-Oriented Approach Based Innovation Workshop in Specialized Training for Preventing Inpatient Falls, Journal of Nursing Management. (2024) 24, no. 3, 185–190.

[32] Yang H. , Zhu H. , Luo W. , and Peng W. , Design and Practice of Innovative Practice Workshop for New Nurses Based on Creativity Component Theory and Outcome Based Education (OBE) Concept, BMC Medical Education. (2023) 23, no. 1, 10.1186/s12909-023-04684-5. PMC10523681 37752513

[33] Guo Y. P. , Lyu X. C. , Zhang C. H. et al., Current Status and Influencing Factors of Proactive Innovation Behavior Among Specialist Nurses, Journal of Nursing Science. (2025) 40, no. 15, 54–57.

[34] Li X. D. , Li Y. , Yang Y. et al., The Influence of Work Environment and Sense of Occupational Benefit on Nurses’ Innovative Behavior, China Health Industries. (2025) 22, no. 18, 45–48.

[35] Yuan Y. , Zhang C. L. , Zhang X. M. et al., Research Progress on Interventions for Clinical Nurses’ Innovation Behavior, West China Medical Journal. (2024) 39, no. 4, 665–670.

[36] Rogers E. M. , Diffusion of Innovations, 2003, 5th edition, Free Press.

[37] Song Y. , Huang M. X. , Liu F. et al., Empirical Study on User Adoption Intention in Early Market of Emerging Technology Products, Studies in Science of Science. (2012) 30, no. 10, 1546–1557+1578.

