# Supplementary material for: Effects of ‘4P’ Theory–Based Training on Innovative Capability in Clinical Nurses: A Pre–Post Study
Source: J Nurs Manag. 2026 Sep 2;2026:2704124. doi: 10.1155/jonm/2704124 (PMC13535847; doi:10.1155/jonm/2704124)
Supplement: Supplementary file 1 [file JONM-2026-2704124-s001.docx]

The outcomes for the Proactive Innovation Group (n=61) and the High-Efficiency Innovation Group (n=61) assessed at baseline and after the one-year intervention are shown in Table S1. In the High-Efficiency Innovation Group, differences in all dimensions except Innovative press were statistically significant (P < 0.05). In the Proactive Innovation Group, differences in all dimensions except Innovative Product were statistically significant (P < 0.05).

Table S1. Changes in Innovation capability Scores Before and After the Intervention in the Two Groups (n=61 per group)

| Item | Group | Pre-test,  M ± SD | Post-test,  M ± SD | Change  (Post‑test – Pre‑test),  M ± SD | Confidence Interval  (95% CI) | T  value | *P* value |
| --- | --- | --- | --- | --- | --- | --- | --- |
| Total innovation capability score | Proactive Innovation Group | 126.67 ± 11.18 | 132.57 ± 8.89 | 5.902±3.29 | [5.06,6.74] | 14.032 | <.001 |
|  | High-Efficiency Innovation Group | 153.33 ± 9.49 | 161.43 ± 9.23 | 8.10 ± 1.74 | [7.65,8.54] | 36.375 | <.001 |
| Innovative person | Proactive Innovation Group | 51.46 ± 5.82 | 53.39 ± 5.31 | 1.934±4.976 | [0.66,3.21] | 3.036 | 0.004 |
|  | High-Efficiency Innovation Group | 63.39 ± 5.98 | 66.98 ± 5.94 | 3.59 ± 5.09 | [2.29,4.90] | 5.505 | <.001 |
| Innovative process | Proactive Innovation Group | 35.89 ± 4.15 | 37.30 ± 3.26 | 1.410±4.165 | [0.34,2.48] | 2.644 | 0.010 |
|  | High-Efficiency Innovation Group | 45.10 ± 5.02 | 47.98 ± 3.87 | 2.89 ± 4.27 | [1.79,3.98] | 5.282 | <.001 |
| Innovative press | Proactive Innovation Group | 25.00 ± 5.22 | 27.92 ± 3.11 | 2.918±5.229 | [1.58,4.26] | 4.358 | <.001 |
|  | High-Efficiency Innovation Group | 28.64 ± 3.80 | 29.10 ± 4.38 | 0.46 ± 5.67 | [-0.99,1.91] | 0.633 | 0.529 |
| Innovative products | Proactive Innovation Group | 14.16± 2.66 | 14.84 ± 3.06 | 0.672±3.78 | [-0.30,1.64] | 1.389 | 0.170 |
|  | High-Efficiency Innovation Group | 16.92 ± 3.13 | 18.02 ± 2.72 | 1.10 ± 4.11 | [0.04,2.15] | 2.085 | 0.041 |
| number of patents obtained | Proactive Innovation Group | 1.00 ± 0.00 | 1.69 ± 0.47 | 0.689±0.467 | [0.570,0.81] | 11.517 | <.001 |
|  | High-efficiency Innovation Group | 3.11 ± 1.59 | 3.98 ± 2.33 | 0.87 ± 0.85 | [0.65,1.09] | 8.020 | <.001 |
| Number of published papers | Proactive Innovation Group | 0.79 ± 0.66 | 1.16 ± 0.76 | 0.377±0.489 | [0.25,0.50] | 6.026 | <.001 |
|  | High-efficiency Innovation Group | 3.90 ± 2.35 | 5.36 ± 5.50 | 1.46 ± 3.57 | [0.55,2.37] | 3.195 | 0.002 |
